# Flavokawain A alleviates the progression of mouse osteoarthritis: An *in vitro* and *in vivo* study

**DOI:** 10.3389/fbioe.2022.1071776

**Published:** 2022-12-05

**Authors:** Shaoze Jing, Junlai Wan, Tianqi Wang, Zhiyi He, Qing Ding, Gaohong Sheng, Shanxi Wang, Hongqi Zhao, Ziqing Zhu, Hua Wu, Wenkai Li

**Affiliations:** ^1^ Department of Orthopedics, Tongji Hospital, Tongji Medical College, Huazhong University of Science and Technology, Wuhan, China; ^2^ Third Hospital of Shanxi Medical University, Shanxi Bethune Hospital, Shanxi Academy of Medical Sciences, Tongji Shanxi Hospital, Taiyuan, China

**Keywords:** flavokawain A, osteoarthritis, apoptosis, autophagy, 3-methyladenine, MAPK, PI3K/AKT/mTOR

## Abstract

Osteoarthritis (OA) is one of the most prevalent chronic degenerative joint diseases affecting adults in their middle or later years. It is characterized by symptoms such as joint pain, difficulty in movement, disability, and even loss of motion. Moreover, the onset and progression of inflammation are directly associated with OA. In this research, we evaluated the impact of Flavokawain A (FKA) on osteoarthritis. *In-vitro* effects of FKA on murine chondrocytes have been examined using cell counting kit-8 (CCK-8), safranin o staining, western blot, immunofluorescence staining, senescence β-galactosidase staining, flow cytometry analysis, and mRFP-GFP-LC3 adenovirus infection. An *in-vivo* model of destabilization of the medial meniscus (DMM) was employed to investigate FKA’s effect on OA mouse. An analysis of bioinformatics was performed on FKA and its potential role in OA. It was observed that FKA blocked interleukin (IL)-1β-induced expression of inflammatory factors, i.e., cyclooxygenase-2 (COX2) and inducible nitric oxide synthase (iNOS) in chondrocytes. In addition, FKA also downregulated the catabolic enzyme expression, i.e., aggrecanase-2 (ADAMTS5) and matrix metalloproteinases (MMPs), and helped in the upregulation of the anabolic protein expression, i.e., type II collagen (Col2), Aggrecan, and sry-box transcription factor 9 (SOX9). Moreover, FKA ameliorated IL-1β-triggered autophagy in chondrocytes, and it was observed that the FKA causes anti-inflammatory effects by the mitogen-activated protein kinase (MAPK) and phosphoinositide-3-kinase/Akt/mammalian target of rapamycin (PI3K/AKT/mTOR) signaling pathways inhibition. The results of immunohistochemical analysis and microcomputed tomography from the *in vivo* OA mouse model confirmed the therapeutic effect of FKA. Finally, we assessed the anti-arthritic impacts of FKA by conducting *in vivo* and *in vitro* analyses. We concluded that FKA can be employed as a useful therapeutic agent for OA therapy, but the findings require needs further clinical investigation.

## Introduction

Osteoarthritis (OA) is one of the most prevalent joint diseases around the globe. Around 18% of women and 10% of men over 60 years of age are affected by it (Woolf and Pfleger, 2003). The most common clinical features of OA are joint pain, swelling, stiffness, and subchondral bone sclerosis (Aicher and Rolauffs, 2014). Non-steroidal anti-inflammatory drugs (NSAIDs) are the main drug of choice for the conservative treatment of OA. But NSAIDs have some significant side effects, including gastrointestinal irritation, ulceration and bleeding (Katz et al., 2021). The treatment of end-stage OA is knee arthroplasty, but the functional results may be poor, and the life of the prosthesis is limited (Glyn-Jones et al., 2015). Therefore, the focus of OA research has shifted to prevention and early treatment. Drug development for joint protection interventions is in progress. Cartilage metabolic imbalance and chronic inflammatory responses are critical in the progression of OA (Appleton, 2018). Increased levels of the proinflammatory cytokine IL-1β, which promotes the cartilage matrix catabolic enzyme synthesis, such as ADAMTS5 and MMPs (Le Maitre et al., 2007), and suppresses the levels of important extracellular matrix (ECM) proteins, including Col2 and Aggrecan (Kobayashi et al., 2005), were detected in the synovial fluid of individuals suffering from OA (Chen et al., 2020). Therefore, drugs that reverse the IL-1β′s effect can help in designing a potential therapy for OA.

In recent years, plant extracts have been recognized for their pharmaceutical significance. Flavokawain A (FKA) is a natural chalcone extracted from Piper methysticum Forst, which has exhibited anti-apoptotic and anti-inflammatory effects (Rajendran et al., 2021). The *in vitro* studies have revealed that FKA inhibits the NFκB and MAPK pathways to relieve lipopolysaccharide-induced inflammation of Raw 264.7 macrophages (Kwon et al., 2013). Furthermore, the ability of FKA to regulate oxidative stress has been published (Hseu et al., 2019). However, the role of FKA in OA has not yet been investigated. Hence, the objective of this research is to elucidate the possible role of FKA in OA treatment.

## Materials and methods

### Ethics approval

For the purpose of minimizing animal suffering, the research was conducted as per the guiding principles and protocols established by the Animal Care and Use Committee for Teaching and Research at the Tongji Medical College of Huazhong University of Science and Technology.

### Chemicals and materials

FKA (T3S0737, purity: 99.41%) and 3-Methyladenine (3 MA) were acquired from TOPSCIENCE (Shanghai, China), and the mouse IL-1β cytokine was obtained from the R&D system (501-RL-010, United States). Safranin O solution were provided by Solarbio (Beijing, China). The primary antibodies for iNOS, COX2, Col2, Aggrecan, Sox9, and immunofluorescence secondary antibodies were acquired from Abcam (Shanghai, China). Atg12, Beclin, LC3I/II, P21, P16, Bax, Bcl2, GAPDH, and antibodies for all pathways were purchased from CST (Beverly, MA, United States). Corresponding primary antibodies for MMP3/13 and Nrf2/HO-1/NQO1 were supplied by the Proteintech Group (Wuhan, Hubei, China). ADAMTS5 primary antibody, secondary antibody, phosphate-buffered saline (PBS), trypsin, collagenase type II, the CCK8 assay kit, bovine serum albumin (BSA), and protein extraction kit were ordered and acquired from Boster Biological Technology (Wuhan, Hubei, China). RFP-GFP-LC3-adenovirus was purchased from Hanbio (Shanghai, China). The Senescence β-galactosidase staining kit and the Annexin V-FITC apoptosis detection kit were supplied by Beyotime (Shanghai, China).

### Isolation and culture of chondrocytes

In our study, five-day-old male C57BL/6J mice were sacrificed to obtain chondrocytes. The cartilage was obtained from around the knee joint region of the mice and was cut into granules after removing the surrounding synovial tissue. The granules were digested with trypsin for half an hour. Later, 0.2% type II collagenase was added for digestion for a period of 6 h. Subsequently, the cell suspension was concentrated by the process of centrifugation at 300g for a period of 5 min to obtain primary chondrocytes. Moreover, the incubation of primary chondrocytes was performed at a temperature of 37°C, with 5% CO_2_ in DMEM/F12 (1:1) medium containing penicillin/streptomycin solution (1%) and FBS (10%). The cells of the third passage were utilized for subsequent experiments.

### Cell viability assay

For the measurement of the viability of cells, the CCK8 assay was employed. Mouse chondrocytes were seeded at a density of 1×10^4^ cells/well in a 96-well plate containing the required culture medium. Following cell adhesion, 0.1 ml of medium with or without IL-1β (5 ng/ml) was added at various concentrations of FKA (0, 5, 10, 20, and 40 μM) for a period of 24 h. Subsequently, an addition of 10 μl of CCK 8 solution was carried out in all the wells, and incubation was performed for a period of 1 h. Thereafter, we employed a microplate reader (Bio-Rad, Richmond, CA, United States) to measure the viability of cells by measuring the absorbance at 450 nm.

### Safranin O staining

Seeding of chondrocytes was performed in 24-well plates. When chondrocytes reached 80% confluency, FKA with a concentration of 40 μM was administered alone or in combination with IL-1β (5 ng/ml). Cells were incubated for 24 h and washed with PBS. Cells were then fixed with 4% paraformaldehyde, and after discarding the cell fixative, they were stained with 0.5% Safranin O reagent for 2 h. The dye was finally removed and washed once again with distilled water.

### Western blot

The protein extraction was performed by first washing the mouse chondrocytes seeded in a 6-well plate using PBS thrice, followed by the addition of 100 μl RIPA lysate buffer, 1 μl phosphatase inhibitor, and protease inhibitor to each well. Additionally, the protein concentration was measured by employing the Bicinchoninic Acid Assay. Moreover, 25 μg of protein samples were added to each well in the SDS-PAGE gel (8.0–12.5%) for electrophoresis. The electroblotting method was employed for the separation of protein bands and transferring onto a polyvinylidene membrane, and blocking of the membrane was performed at room temperature using BSA (5%) for a period of 1 h, and overnight incubation of the target band was carried out in the corresponding primary antibody at a temperature of 4°C. Washing of the membrane was conducted in TBST three times, and a second incubation was performed in the corresponding secondary antibody for a period of 1 h, then rinsed thrice with TBST again. The Image Lab software (Bio-Rad) was used to obtain clear western blotting bands.

### Immunofluorescence

Laser confocal culture dish (34.8 mm diameter) was utilized to seed the chondrocyte cells at a density of 1 × 10^4^ cells/well. 5 ng/ml of IL-1β was added for treating the cells for 24 h in the absence or presence of 40 µM FKA. Furthermore, PBS was utilized for washing (three times); cell fixation was carried out using 4% paraformaldehyde for a period of 15 min; cell permeabilization was performed by employing 0.5% Triton X-100 for a period of 15 min, and 5% BSA was employed for blocking. Overnight incubation of the chondrocytes was done with primary antibody against MMP13 (18165-1-AP, 1:50) and Aggrecan (ab36861, 1:100) at 4°C. After that, they were rinsed using PBS three times, and incubation was performed again with fluorescent secondary antibodies (ab150083, 1:200) for a period of 60 min. In addition, 0.5 μg/ml DAPI was utilized for the nuclei staining. The cell imaging was carried out with the aid of a confocal microscopy (Leica, United States).

### Senescence β-galactosidase staining

The senescent β-galactosidase staining was utilized to check the degree of cellular senescence. The washing of cells was conducted using PBS, and fixation was carried out for 15 min. After that, the staining solution was added for overnight incubation at 37°C in the absence of carbon dioxide. A microscope was employed for the observation and recording of the number of blue spots in the field of view.

### Flow cytometry analysis

Chondrocytes were collected using 0.1% trypsin following the instructions provided by the manufacturer; PBS was used for washing (twice), and cell resuspension was performed in a binding buffer. Apoptotic cells were assessed by flow cytometry (Beckman Coulter, Inc. United States) after performing staining using propidium iodide and Annexin V (Sun et al., 2021).

### Detection of autophagic flux

RFP-GFP-LC3-adenovirus was employed for the detection of autophagic flux. When the cells reached about 50% confluency, the cells were infected with adenovirus vectors for a day. Then use IL-1β (5 ng/ml) pretreated cells in the presence or absence of FKA (40 μM) for 24 h. Due to acid sensitivity, green fluorescence is extinguished after autolysosome formation, and only red fluorescence can be detected (Pei et al., 2021). The number of autophagosomes and autolysosomes was then observed by confocal microscopy (Leica, United States).

### Bioinformatics analysis of FKA

Through the PharmMapper and miRWalk2.0 databases, the FKA and OA-related pathways were identified, respectively. The prediction of FKA-target genes was performed based on the PharmMapper database, which was subsequently imported into the Database for Annotation, Visualization, and Integrated Discovery (DAVID) to carry out the Kyoto Encyclopedia of Genes and Genomes (KEGG) pathway enrichment analysis. Moreover, in order to obtain FKA-related OA pathways, the incorporation of the KEGG pathways was conducted with OA-related pathways acquired using the miRWalk 2.0 database. Additionally, the Venn diagram (Venny2.1, http://bioinfogp.cnb.csic.es/tools/venny/index.html) was utilized for the visualization of various intersected pathways related to FKA. Furthermore, Cytoscape 3.8.2 was employed for the screening of hub genes based on the degree, betweenness, and closeness centrality of FKA-target genes. The Pathway Builder Tool 2.0 (https://www.proteinlounge.com) was employed to conduct KEGG pathway enrichment analysis, and the leading KEGG pathways were selected from the crossover pathways. Finally, the proximity of FKA-target genes in the network were illustrated by constructing a circos plot with the aid of the circlize R package, which also included the location of the genes on the chromosome and their connections.

### DMM OA model

Eighteen specific pathogen-free C57BL/6J male mice were acquired from the Experimental Animal Center of Tongji Hospital. Mouse DMM OA model was established as previously described (Lu et al., 2021). The mouse were anesthetized by intraperitoneal injection of pentobarbital (100 μl/10g body weight). Right knee surgery of all the mice was carried out and randomly classified into 3 groups. FKA group mice (*n* = 6) underwent DMM surgery and were intra-articularly injected with 50 mg/kg FKA weekly. Sham-operated group mice (*n* = 6) underwent sham surgery (capsulotomy and suturing) and were injected with saline (equal volume). OA group mice (*n* = 6) underwent DMM surgery and were injected intra-articularly with saline (equal volume). After Eight weeks of surgery, all mice were sacrificed with overdose anesthetics, and sample collection and fixation were performed using 4% paraformaldehyde for 24 h.

### Histological evaluation

Decalcification of the fixed samples was performed for 30 days using the EDTA-Decalcifying-fluid, and 5-μm-thick fragments of the right knee joint were cut. In addition, hematoxylin and eosin (H&E), safranin-o-fast green as well as toluidine blue were used for the staining of these fragments (Pan et al., 2022). The expression of Aggrecan and Col2 in mouse cartilage was assessed by immunohistochemistry. The assessment of the changes in mouse articular cartilage was carried out as per the Osteoarthritis Research Society International (OARSI) scoring system (Glasson et al., 2010). Scoring was done by two independent investigators who have no knowledge of the grouping.

### Micro-computed tomography

We used a small animal micro-CT (Scanco Medical AG, Bassersdorf, Switzerland) for the purpose of scanning the right knee joint of the mice. After obtaining the image, the internal software of the microCT system was used for 3D reconstruction. Statistical analysis was then performed using the trabecular numbers (Tb.N), trabecular separation (Tb.Sp), and bone volume/tissue volume (BV/TV) of the samples.

### Statistical analysis

Independent repetition of each experiment was conducted at least thrice, and analysis of data was carried out by employing the GraphPad prism V.8 software (GraphPad Inc., La Jolla, CA, United States). Additionally, all the data were represented by mean +standard deviation (SD), and relevant analyses were conducted using one-way analysis of variance (ANOVA) followed by Turkey’s post-hoc test. Statistical significance was determined by *p* ≤ 0.05.

## Results

### Effect of FKA on cell viability

**FIGURE 1:**
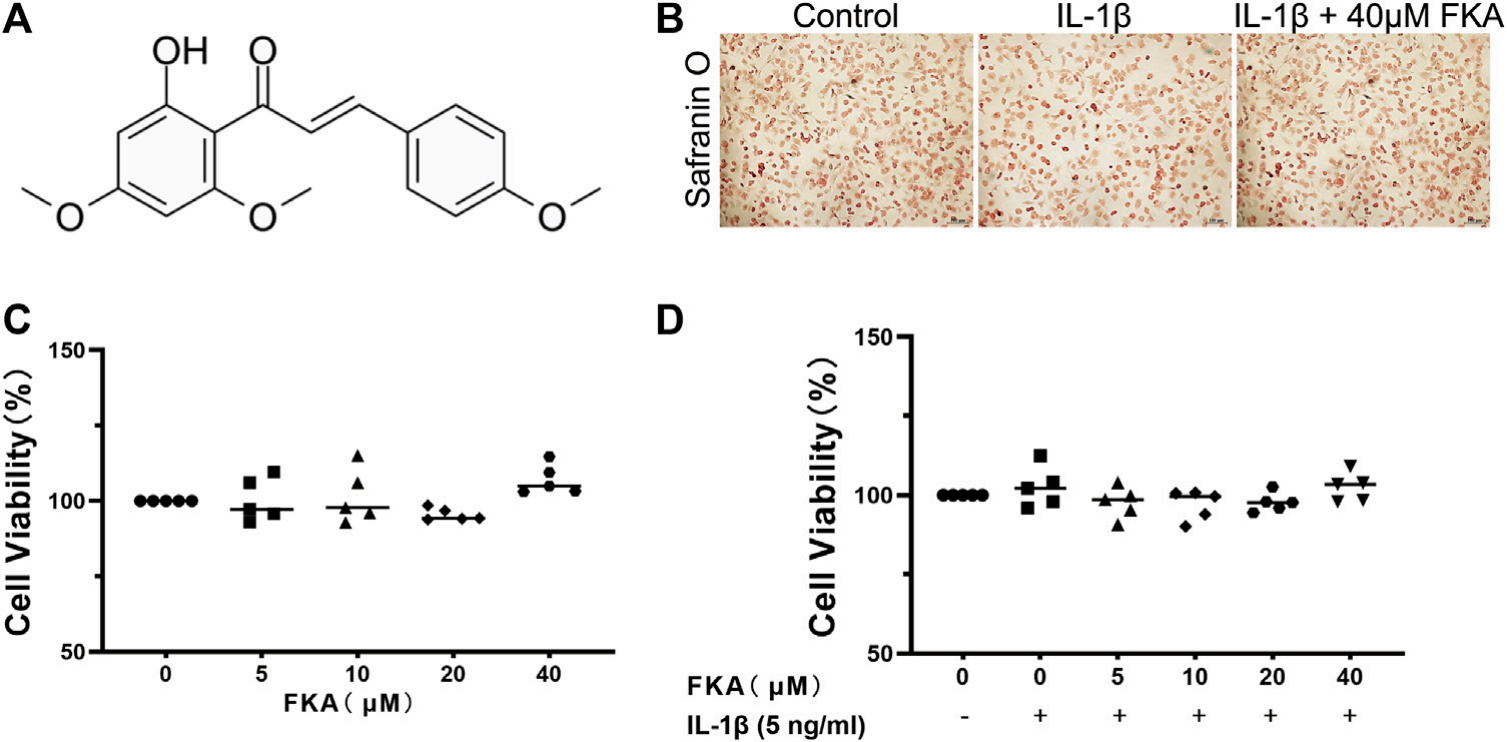
Effects of FKA on chondrocyte viability **(A)** Chemical Structure of FKA. **(B)** Safranin O staining of chondrocyte cells after treatment with IL-1β (5 ng/ml) and FKA for a period of 24 h (scale bar 100 μm). Viability of chondrocyte cells after 24 h intervention with various concentrations of FKA in the absence **(C)** or presence of IL-1β **(D)** tested using the CCK8 kit. The values are presented as means ± SD (*n* = 3).

We used the CCK 8 kit to determine the impact of various concentrations of FKA on the viability of chondrocytes. The molecular structure of FKA can be seen in Figure 1A. The illustrations in Figures 1C,D reveal that the presence or absence of IL-1β (5 ng/ml) and different concentrations of FKA had no effect on cell viability. Based on current and previous findings (Kwon et al., 2013), we used 20 μM and 40 μM of FKA for subsequent experiments. In addition, Safranin O staining showed that the morphology of chondrocytes did not change after FKA intervention (Figure 1B).

### FKA inhibits chondrocyte inflammation and catabolism induced by IL-1β

**FIGURE 2:**
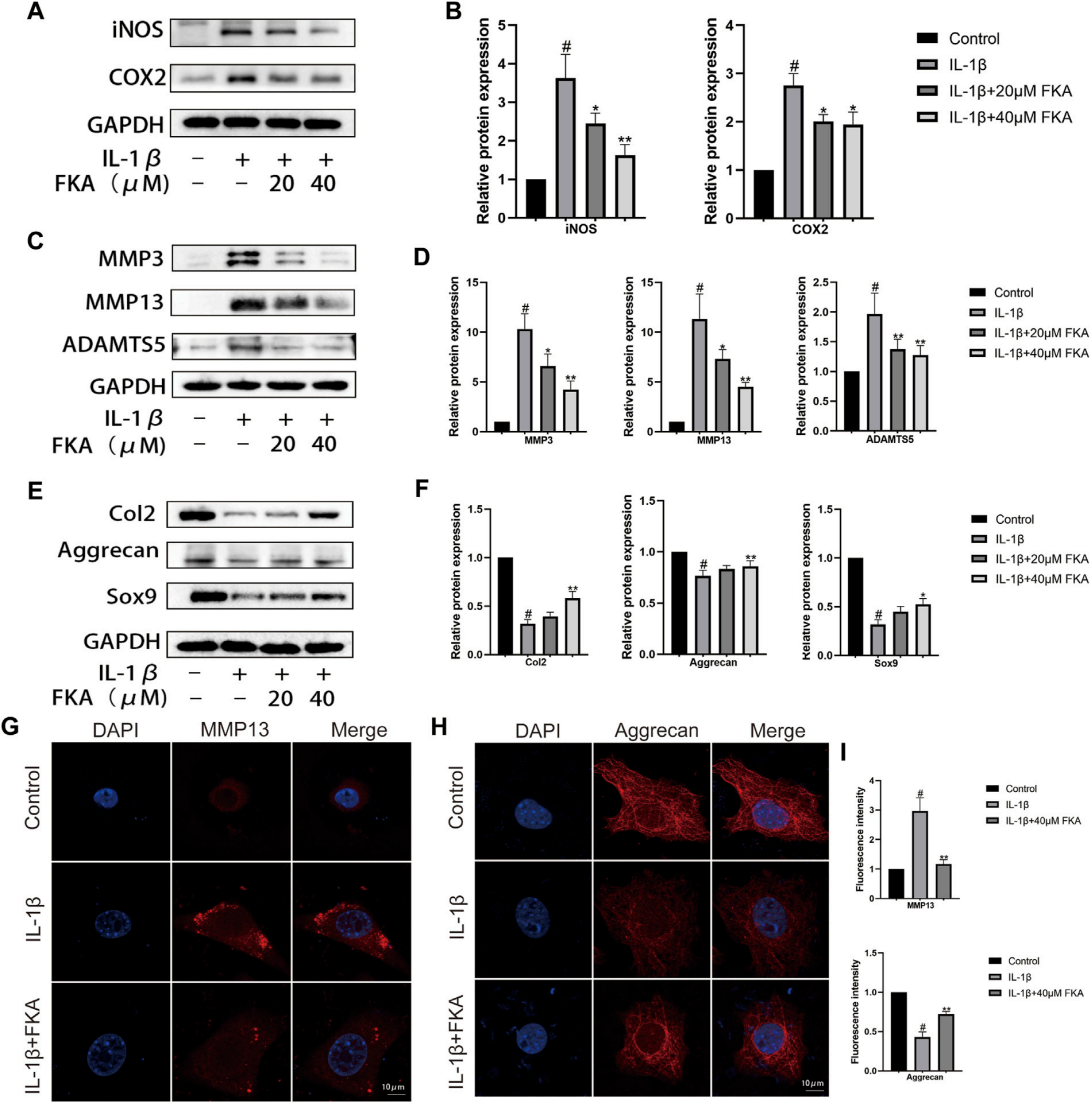
Protective effects of FKA against IL-1β-induced inflammation and ECM destruction in mouse chondrocytes. Chondrocyte cell treatment with 5 ng/ml of IL-1β only or with FKA (20 and 40 μM) for a period of 24 h. Western blot analysis of inflammatory cytokines (COX2 and iNOS), catabolic (ADAMTS5, MMP3, and MMP13), and anabolic markers (Col2, Aggrecan, and Sox9) **(A,C and E)**. Quantitative analysis of protein expression **(B,D and F)**. **(G and H)** 5 ng/ml of IL-1β was added for treating the cells for 24 h in the absence or presence of 40 µM FKA. Confocal microscopy showing the expression of MMP13 and Aggrecan in immunofluorescence experiments (scale bar 10 μm). **(I)** Relative quantification of fluorescence intensity. The values are presented as means ± SD (*n* = 3). #*p* < 0.05 vs the control group; *p < 0.05 and **p < 0.01 vs the IL-1β group.

The western blot was utilized to assess the impact of FKA on cartilage inflammation and metabolism. Figure 2(A–D) illustrates that IL-1β elevated the expression levels of chondrocyte inflammatory cytokines (COX2 and iNOS), MMPs (MMP3/13), and ADAMTS5, whereas FKA lowered their expression levels in a concentration-dependent manner. Additionally, FKA also reversed the impacts of IL-1β on the anabolism of chondrocytes. The expression of Col2, Aggrecan, and Sox9 in chondrocytes showed a significant downward trend under the influence of IL-1β, but the effect was reversed under the effect of FKA (Figures 2E,F). The results of immunofluorescence were consistent with the western blot data (Figures 2G–I).

### FKA regulates the expression of senescence-related genes in chondrocytes

**FIGURE 3:**
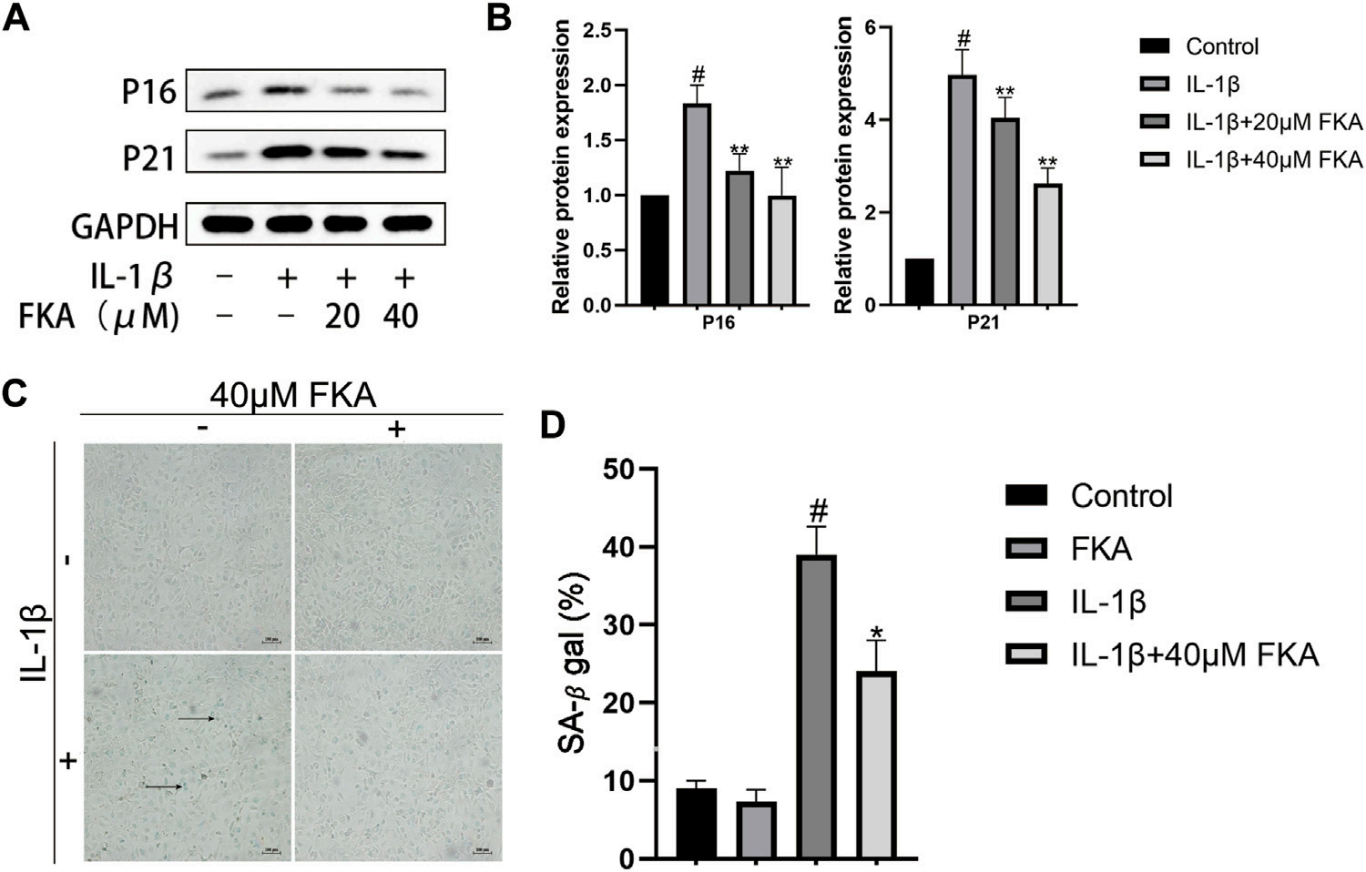
The regulation of FKA on the expression of senescence-related genes in chondrocytes **(A)** Western blot analysis revealing P16 and P21 expression in chondrocytes. **(B)** Quantitative analysis of protein expression **(C)**. β-Galactosidase used for the staining of chondrocyte cells (scale bar 100 μm) **(D)** Quantitative analysis of β-galactosidase expression. The values are presented as means ± SD (*n* = 3). #*p* < 0.05 vs control group; *p < 0.05 and **p < 0.01 vs IL-1β group.

The chondrocyte cells during OA exhibited elevated levels of various senescence markers, for instance, β-galactosidase, as well as the increased expression of senescence-related genes (McCulloch et al., 2017). Figures 3A,B show that the levels of expression of P16 and P21 proteins were elevated in chondrocytes under the influence of IL-1β, whereas FKA suppressed their expression. Additionally, the senescence β-galactosidase staining kit was employed to test the β-galactosidase activity in different groups. Under elevated IL-1β levels, the activity of β-galactosidase was increased (more blue spots), and FKA decreased its activity (Figures 3C,D).

### FKA suppresses IL-1β-induced apoptosis in mouse chondrocytes

**FIGURE 4:**
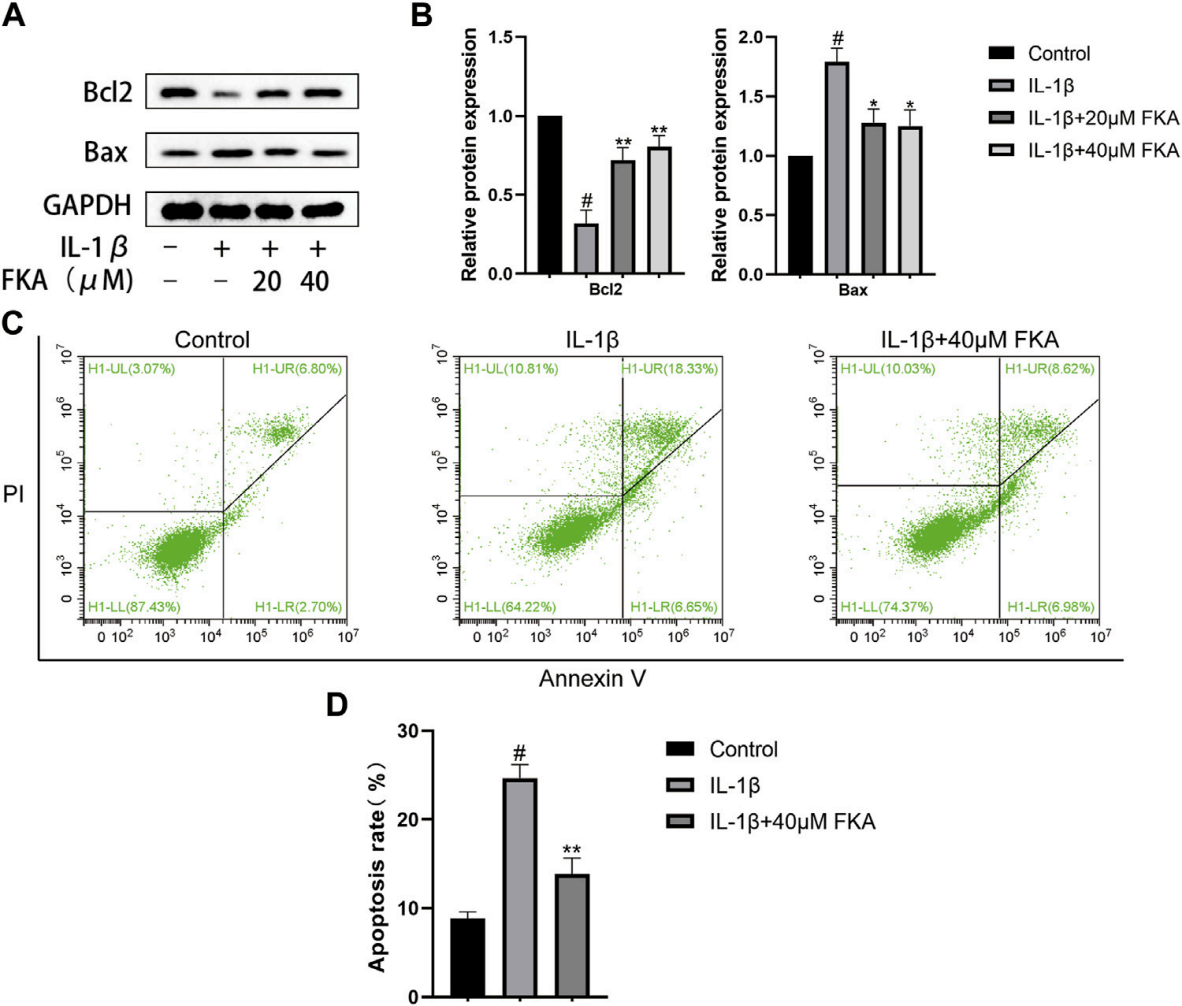
Impacts of FKA on IL-1β-induced apoptosis in mouse chondrocytes **(A)** Western blot findings revealing Bax and Bcl2 expression in chondrocytes. **(B)** Quantitative analysis of protein expression. **(C)** The apoptosis rate of chondrocyte cells was detected by flow cytometry. **(D)** The apoptosis rate of chondrocyte cells in all groups were detected with Annexin V-FITC/PI staining and flow cytometry. The values are presented as means ± SD (n = 3). #*p* < 0.05 vs control group; *p < 0.05 and **p < 0.01 vs IL-1β group.

Chondrocyte apoptosis has a close correlation with the severity of cartilage damage in OA. In the current study, we treated chondrocytes with FKA in the IL-1β presence or absence for a period of 24 h. Figures 4A,B indicate that treatment with IL-1β markedly upregulated Bax and downregulated Bcl2 expression in chondrocytes. However, FKA alleviated these trends. In order to determine the apoptosis status of chondrocytes, we employed the Annexin V-FITC/PI Apoptosis Detection kit. Figures 4C,D illustrate that FKA treatment led to a remarkable decrease in IL-1β-induced apoptosis of chondrocytes.

### FKA regulates IL-1β-induced suppression of the expression of autophagy-related genes in chondrocytes

**FIGURE 5:**
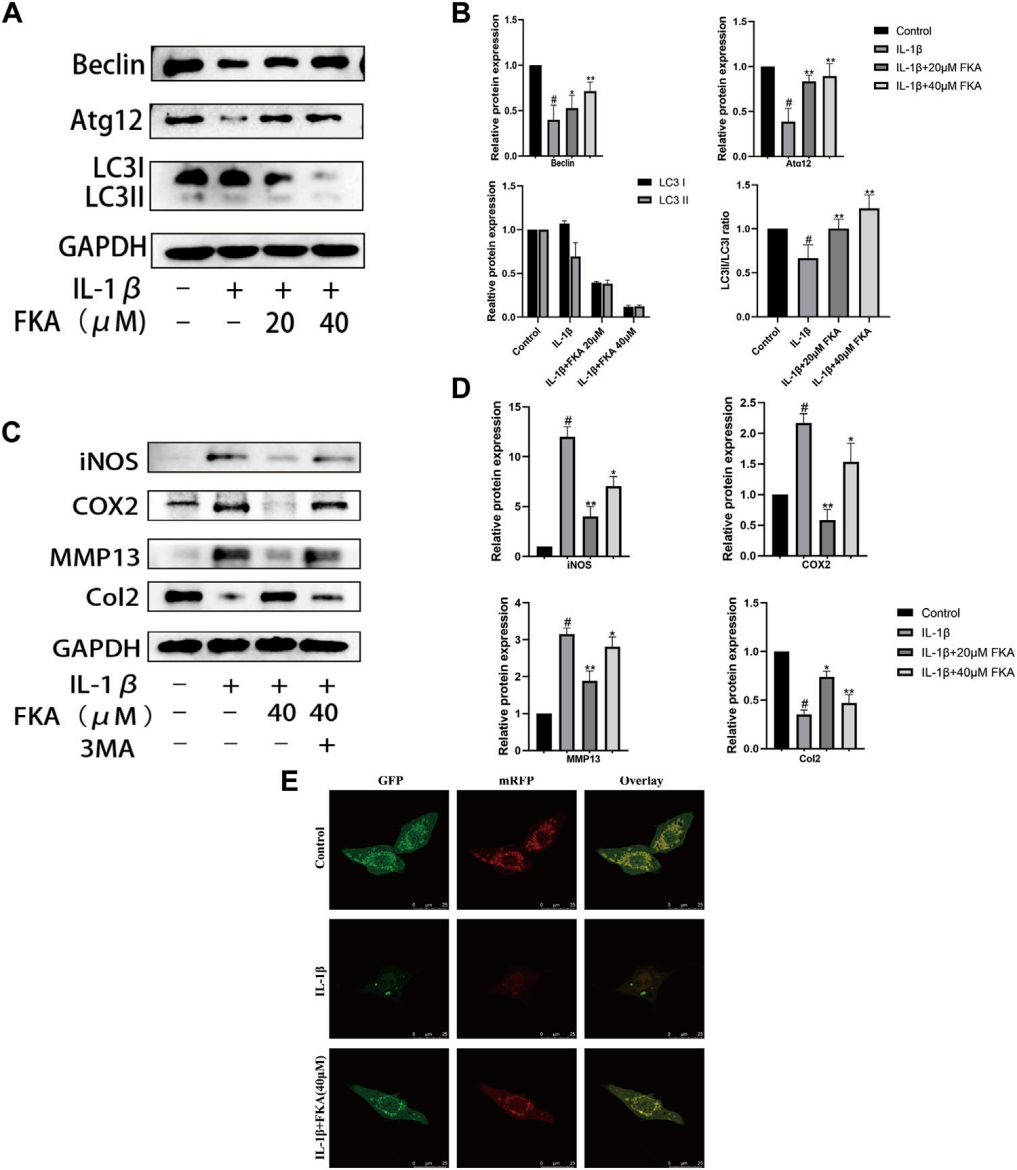
FKA regulates IL-1β-induced autophagy-related gene expression in chondrocytes **(A)** Western blot results show Beclin, ATG12, and LC3I/LC3II expression in chondrocytes. **(B)** Quantitative analysis of protein expression. **(C)** Effect of FKA on autophagy in chondrocytes after treatment with the autophagy inhibitor 3 MA. **(D)** Quantitative analysis of protein expression. **(E)** After 5 ng/ml of IL-1β was added for treating the cells for 24 h in the absence or presence of 40 µM FKA, detection of autophagy in chondrocytes transfected with RFP-GFP-LC3-adenovirus was performed (scale bar 25 μm). Autophagosomes denoted by the green dots, autophagolysosomes denoted by the red dots. The values are presented as means ± SD (*n* = 3). #*p* < 0.05 vs control group; *p < 0.05 and **p < 0.01 vs IL-1β group.

Numerous pieces of evidence indicate that a crucial role is played by autophagy in the progression of OA (Musumeci et al., 2015; Liu et al., 2021; Yan et al., 2022). Therefore, we analyzed the impacts of FKA on autophagy in IL-1β-treated mouse chondrocyte cells. Figures 5A,B reveal that IL-1β treatment remarkably suppressed the ATG12, LC3Ⅱ/LC3, and Beclin, expression levels in mouse chondrocytes. However, the administration of 40 μM FKA significantly reversed these changes. Moreover, RFP-GFP-LC3-adenovirus was employed to further verify the impact of FKA on autophagy. We observed that the number of autophagolysosomes and autophagosomes decreased in chondrocyte cells treated with IL-1β, and FKA pretreatment reversed this trend as illustrated in Figure 5E. Furthermore, the autophagy inhibitor, 3MA, attenuated the promoting effect of FKA on IL-1β-induced chondrocyte autophagy (Figures 5C,D).

### FKA alters antioxidant gene expression in chondrocytes

**FIGURE 6:**
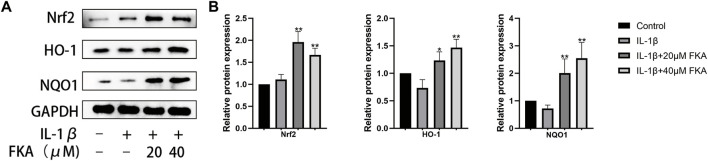
FKA alters antioxidant gene expression in chondrocytes **(A)** Western blot assay revealing Nrf2, HO-1, and NQO1 expression in chondrocytes. **(B)** Quantitative analysis of protein expression. The values are presented as means ± SD (n = 3). #*p* < 0.05 vs control group; *p < 0.05 and **p < 0.01 vs IL-1β group.

A crucial role is exerted by oxidative stress in the advancement of OA (Feng et al., 2019). According to a previous study, FKA alters the expression of antioxidant genes in primary splenocytes (Yang et al., 2020). Therefore, we assessed whether FKA could alter the IL-1β-induced expression of antioxidant genes, including NQO1, Nrf2, and HO-1 in chondrocytes. The results in Figures 6A,B illustrate that FKA substantially increased the expression of antioxidant genes in chondrocytes.

## Results of bioinformatics analysis of FKA

**FIGURE 7:**
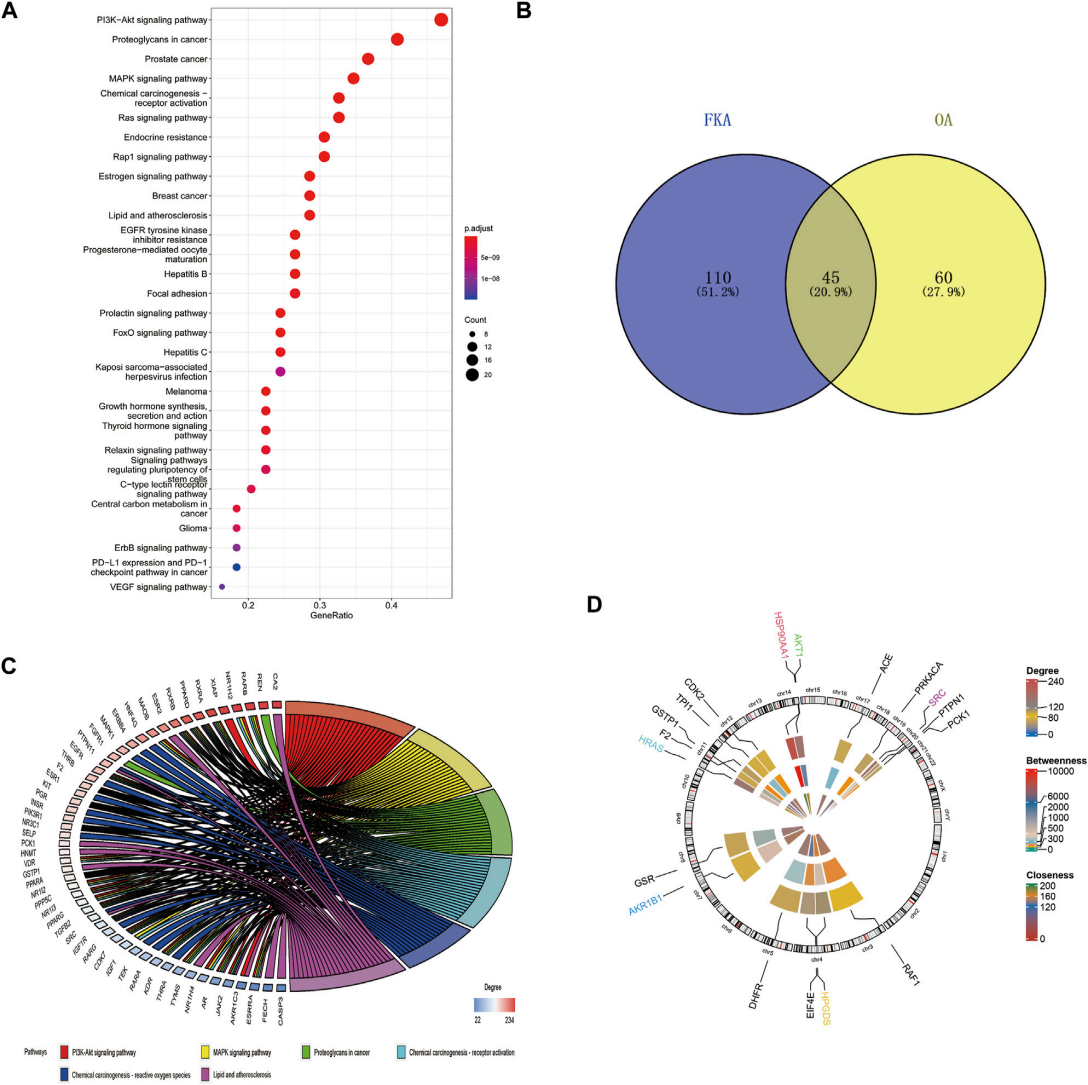
Bioinformatics analysis of FKA pathways and target genes **(A)** KEGG pathway enrichment analysis of FKA target genes. **(B)** The intersection between FKA-related KEGG pathways and OA-related pathways. **(C)** Top six paths in cross paths (KEGG enriched chord diagram). **(D)** Assessment of Centrality and chromosomal investigation of FKA-related central genes.

The identification of potential FKA-target genes was performed using the PharmMapper database. The KEGG pathway enrichment analysis revealed the enrichment of FKA -target genes in 155 pathways. The four leading pathways identified with the most enriched genes were PI3K/Akt, proteoglycans in cancer, prostate cancer, and MAPK signaling pathways (Figure 7A). In addition, 105 OA-related pathways were identified based on the mirWalk2.0 database. We identified 45 common pathways related to FKA and OA (Figure 7B). Information on the six KEGG pathways with the most enriched genes, including the PI3K/Akt, MAPK signaling pathway, proteoglycans in cancer, Chemical carcinogenesis -receptor activation, Chemical carcinogenesis -reactive oxygen species, and Lipid and atherosclerosis is presented in Figure 7C. Figure 7D illustrates the circular diagram revealing the FKA-target genes’ chromosomal position and connectivity. Moreover, the AKT1 gene also illustrates a superior closeness, degree, and betweenness centrality.

### Effect of FKA on the PI3K/Akt/mTOR and MAPK pathways induced by IL-1β in mouse chondrocytes

**FIGURE 8:**
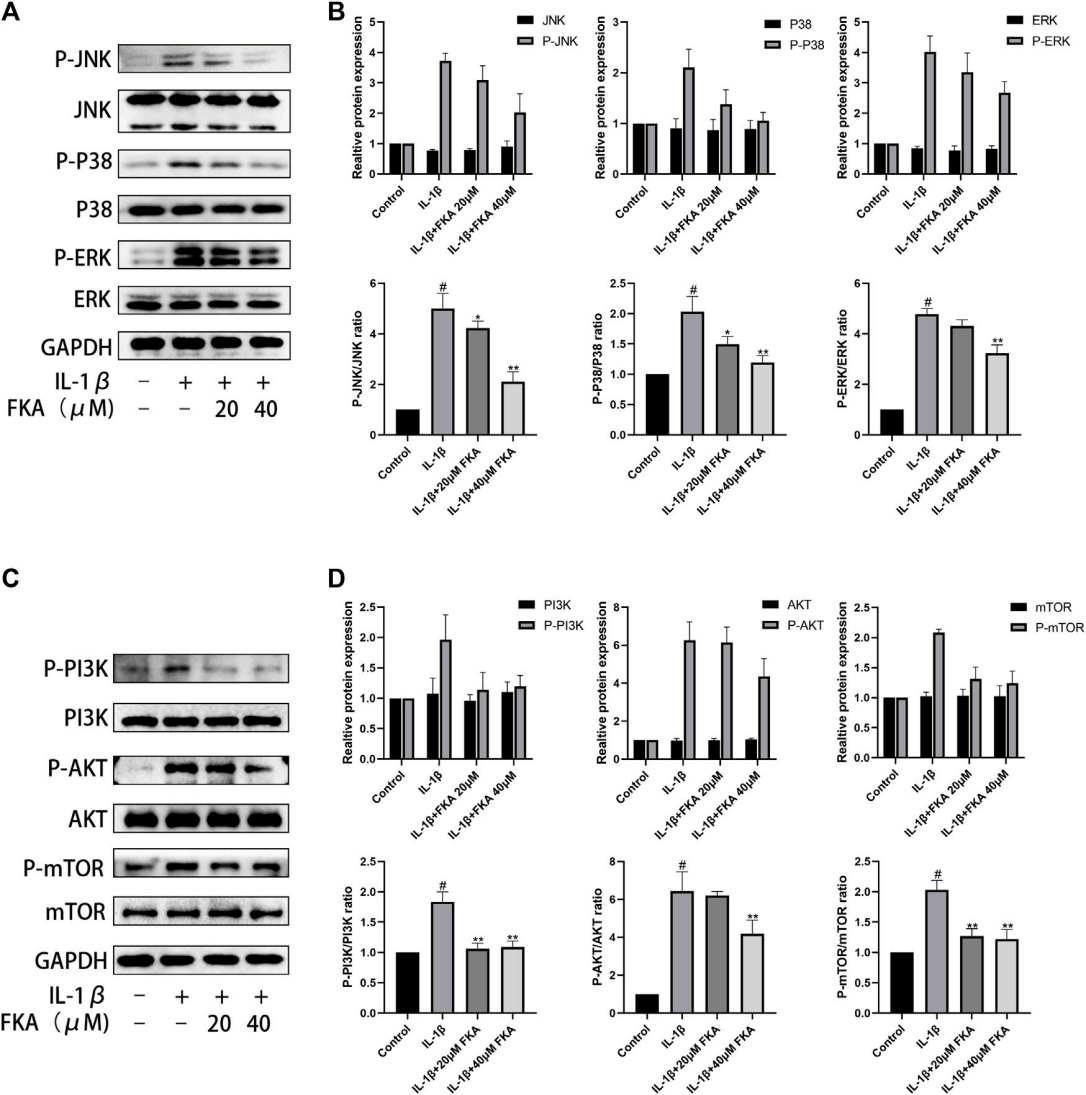
Effect of FKA on the PI3K/Akt/mTOR pathway and MAPK pathway induced by IL-1β in mouse chondrocyte cells **(A)** The expression of proteins of the MAPK pathway illustrated by western blot analysis. **(B)** Quantitative analysis of protein expression. **(C)** Western blot results show the expression of proteins of the PI3K/Akt/mTOR pathway. **(D)** Quantitative analysis of protein expression. The values are presented as means ± SD (*n* = 3). #*p* < 0.05 vs control group; *p < 0.05 and **p < 0.01 vs IL-1β group.

The PI3K/Akt/mTOR and MAPK pathways exert a significant function in the autophagy, inflammation, and progression of OA (Park et al., 2018). In this study, treatment of chondrocyte cells was performed with IL-1β only or in combination with 20 μM or 40 μM FKA for 30 min, and protein expression in chondrocytes was assessed using the western blot technique. As shown in (Figure 8), FKA pretreatment partially inhibited IL-1β-stimulated phosphorylation of the genes involved in the MAPK and PI3K pathways.

### Effects of FKA on mouse OA model

**FIGURE 9:**
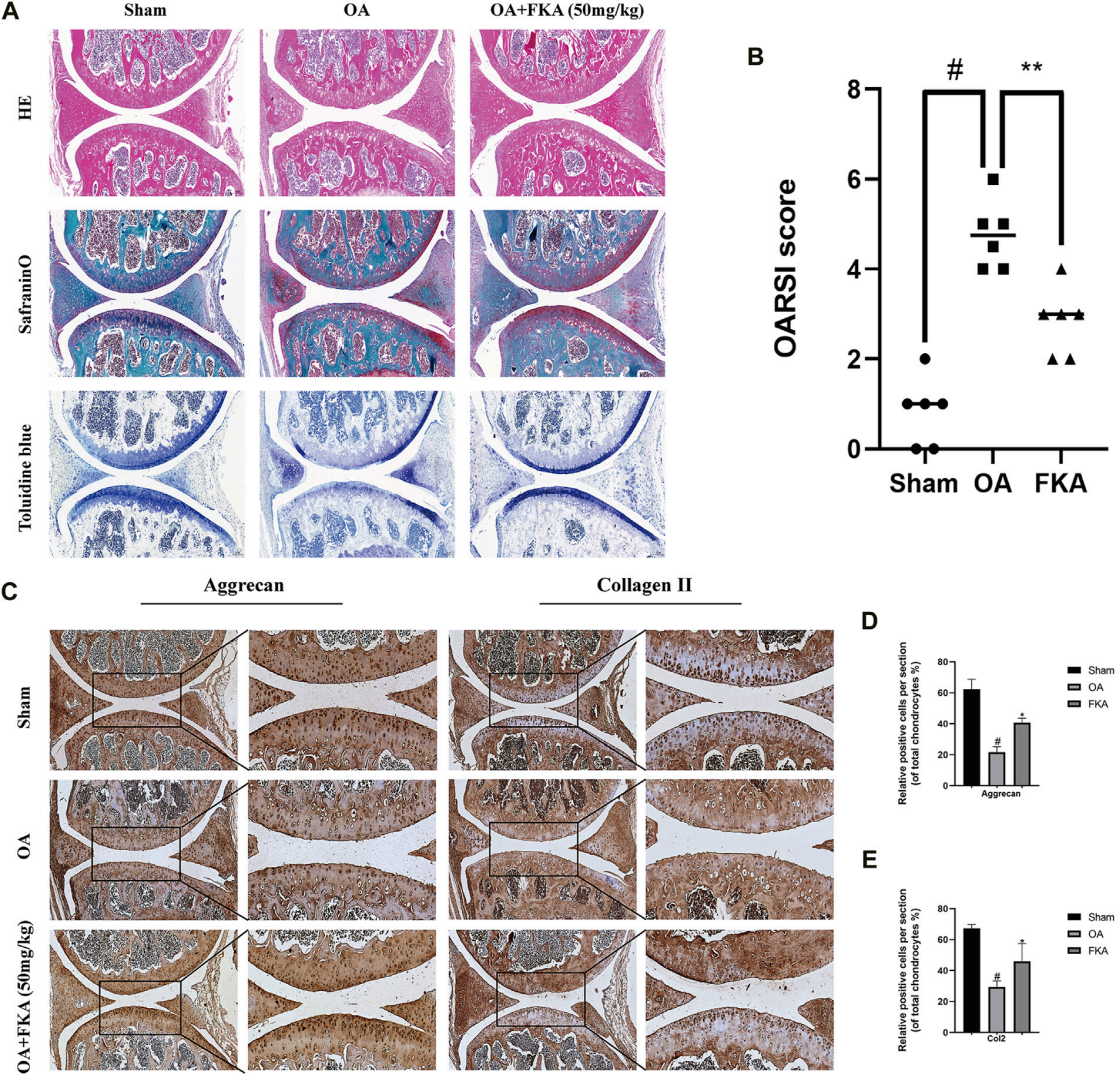
Impacts of FKA on mouse OA model **(A)** H&E, safranin, and toluidine blue staining of cartilage in the mouse OA model. **(B)** Osteoarthritis Research Society International (OARSI) scoring system **(C)** Immunohistochemistry staining of the expression of Aggrecan and Col2 were conducted on three groups of cartilage tissues (scale bars, 50 and 100 μm). **(D and E)** Quantitative analysis of immunohistochemistry. The values are presented as means ± SD (*n* = 6). #*p* < 0.05 vs sham group; *p < 0.05 vs OA group.

Furthermore, the effects of FKA on OA onset and progression were detected in the mouse models. The histological analysis revealed remarkably less cartilage damage in the FKA group in comparison to the OA model group (Figures 9A,B). Moreover, immunohistochemical results revealed that the FKA group showed significantly increased expression of Aggrecan and Col2 in comparison to the OA model group (Figures 9C–E).

### Micro-CT evaluation of the effect of FKA on subchondral bone remodeling in mouse OA model

**FIGURE 10:**
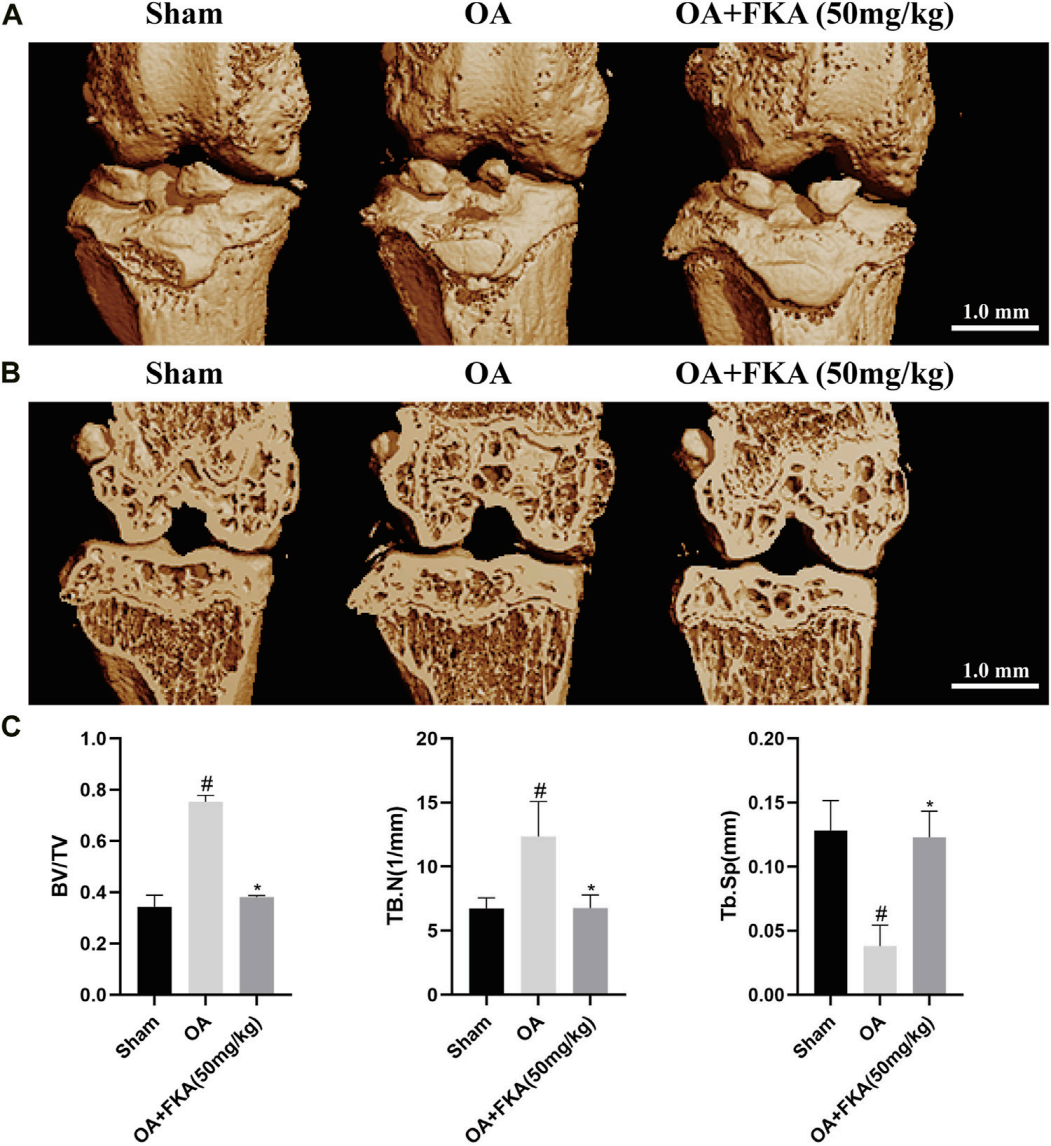
Micro-CT evaluation of the impact of FKA on subchondral bone remodeling in the mouse OA model. **(A)** 3D image of mouse OA model generated by micro-CT scan. **(B)** Coronal image views of the right knee from the sham, OA, and OA+ FKA group mice. **(C)** BV/TV, Tb.N, and Tb. Sp of the three groups. *p < 0.05 and **p < 0.01 vs sham group (scale bar 1 mm).

A micro-CT was conducted for the evaluation of the impact of FKA on subchondral bone remodeling in the OA models. In Figure 10, we can observe that the bone volume fraction (BV/TV) and the trabecular number (Tb.N) in the OA model group were evidently increased in comparison to the sham group, and the degree of trabecular separation (Tb.Sp) was significantly reduced. However, after FKA treatment, these trends were significantly reversed. These findings suggest that FKA had a promising amelioration impact on subchondral bone remodeling in OA mice.

## Discussion

As the existing treatments are unsatisfactory, osteoarthritis will undoubtedly continue to deteriorate the quality of life of the elderly. In recent years, natural plant extracts have attracted a lot of attention in the treatment of diseases (Robinson et al., 2016). FKA, a chalcone from *Piper methysticum* Forst extract, has been reported to have multiple biological activities. In the research conducted by our group, we reported FKA’s therapeutic effects in a mouse model of OA and its underlying mechanism of action. Local inflammatory responses are crucial in the development of OA as they induce the secretion of inflammatory mediators, i.e., TNFα and IL-1β (Dumond et al., 2004). For the activation of different inflammatory pathways, for instance, the PI3K/Akt and MAPK pathways, IL-1β plays a crucial role. Several studies have shown that inhibition of these pathways can contribute to the reduction of inflammation and matrix-degrading enzymes, and to the improvement of cartilage degeneration in the long run (Lianxu et al., 2006; Herrero-Beaumont et al., 2019). We observed that the inflammatory factors such as COX2 and iNOS and catabolic factors, like ADAMTS5, MMP3, and MMP13 significantly increased after IL-1β intervention in chondrocytes, but anabolic enzymes, for instance, Col2, Aggrecan, and SOX9 were significantly reduced. These trends have been reversed with the addition of FKA in a dose dependent manner. Cellular senescence is also an important part of OA progression. Senescent cells in joint tissues are thought to be responsible for the development of OA (Coryell et al., 2021). Our study shows that FKA can substantially suppress the expression of P16 and P21*,* senescence cell-related genes, in chondrocytes induced by IL-1β. OA is also characterized by enhanced apoptosis in chondrocytes (Hwang and Kim, 2015). Therefore, reducing the apoptosis rate of chondrocytes is a potential treatment strategy for OA. We found that the rate of IL-1β-induced apoptosis can be attenuated by FKA in chondrocyte cells. Barranco, (2015) found that the AKT-mTOR signaling pathway directly mediates autophagy in chondrocyte cells. The onset and progression of OA mediated by excessive production of reactive oxygen species (ROS) are countered by autophagy (Charlier et al., 2016). Autophagy decreases cellular ROS, resulting in the protection of chondrocytes. Therefore, a significant role is played by autophagy in the protection of chondrocyte cells from oxidative stress (Ansari et al., 2018). Furthermore, the Nrf2/HO-1 signaling pathway contributes to the resistance to oxidative stress, and its activation can promote the expression of multiple antioxidant factors downstream. In our study, it was confirmed that FKA could partially reverse IL-1β-induced downregulation of autophagy. The protective impact of FKA on IL-1β-induced chondrocyte cells was reduced under the influence of 3MA, an autophagy inhibitor. Moreover, FKA also promoted the Nrf2/HO-1 signaling pathway expression. Therefore, FKA protects against oxidative stress by promoting autophagy and activating anti-oxidative pathways. As a result of bioinformatic analysis, we identified AKT1 as a potential target of FKA. Previous study confirmed that miR-495 induced chondrocyte apoptosis and senescence *via* directly targeting AKT1 and regulating the AKT/mTOR signaling pathway (Zhao et al., 2019). It may be possible in future studies to determine whether FKA acts as an anti-arthritis agent by targeting AKT1. Also, bioinformatic analysis suggested that the PI3K/AKT pathway and MAPK pathway could be two potential mechanisms of FKA involved in the occurrence and progression of OA. These mechanisms are directly linked to the onset and progression of OA (Wu et al., 2021), as well as closely related to autophagy. It has been demonstrated in previous studies that the PI3K/AKT/mTOR and MAPK pathways inhibition can reduce the inflammatory response in OA (Zhou et al., 2019). Our results show that FKA can protect chondrocytes by inhibiting the phosphorylation of the genes involved in PI3K/AKT/mTOR and MAPK pathways induced by IL-1β. In order to validate the therapeutic effects of FKA, we performed *in vivo* experiments. As expected, FKA alleviated the OA progression in a mouse OA model.

In summary, these results emphasize FKA’s potential as a reliable drug for treating OA. However, there are some limitations to this study. Firstly, detrimental concentrations of FKA on chondrocytes were not validated. In addition, the possible effects of FKA on other organs (liver and kidney, *etc.*) were not investigated. In comparison with systemic drug delivery, intraarticular delivery offers numerous advantages, including the increase in local bioavailability, the reduction of adverse events, and the lower cost (Emami et al., 2018). In spite of this, intraarticular therapies remain controversial in terms of their efficacy. The drug residence time in the joint is largely determined by the size of the molecule (Jones et al., 2019). The drug residence time of FKA in the articular cavity has not been studied in this study, which is another limitation. Moreover, ineffectiveness of systemic delivery and low bioavailability of natural plant extracts are also factors that have not yet been translated into clinical applications. During the past decade, remarkable progress has been made in the development of novel drug delivery systems (nanoparticles) for encapsulating plant active metabolites. Hence, combining FKA and novel nanocarriers may be the key to making it applicable in clinical settings, which can be explored in further studies (Bharali et al., 2011; Rahman et al., 2020).

## Conclusion

In conclusion, this report is the first to observe that FKA can protect chondrocytes by inhibiting the MAPK and PI3K pathways. FKA can promote the autophagy of chondrocytes and improve their antioxidant capacity. Our findings offer insights into developing novel treatment strategies for OA.

## Data Availability

The original contributions presented in the study are included in the article/supplementary material, further inquiries can be directed to the corresponding authors.

## Abbreviations

FKA, Flavokawain A; OA, osteoarthritis; COX2, cyclooxygenase-2; IL-1β, interleukin-1 beta; iNOS, inducible nitric oxide synthase; MMPs, matrix metalloproteinases; SOX9, sry-box transcription factor 9; CCK-8, cell counting kit-8; KEGG, Kyoto Encyclopedia of Genes and Genomes; MAPK, mitogen-activated protein kinase; PI3K/AKT/mTOR, phosphoinositide-3-kinase/Akt/mammalian target of rapamycin; HE, hematoxylin and eosin; OARSI, Osteoarthritis Research Society International; Nrf2, Nuclear factor erythroid 2-related factor 2; HO-1, Heme Oxygenase-1 Antibody; NQO1, NAD(P)H:quinone oxidoreductase 1.
